# Influence of TiO_2_ Immobilization Strategy on BC–TiO_2_ Nanocomposite Photocatalytic Performance

**DOI:** 10.3390/ma19173575

**Published:** 2026-08-23

**Authors:** Paul Alcocer, Omar P. Troncoso, Fernando G. Torres

**Affiliations:** Department of Mechanical Engineering, Pontificia Universidad Católica del Perú, Av. Universitaria 1801, Lima 15088, Peru; a20255082@pucp.edu.pe (P.A.); fgtorres@pucp.pe (F.G.T.)

**Keywords:** bacterial cellulose, photocatalytic performance, immobilization strategy, titanium dioxide, water remediation

## Abstract

**Highlights:**

**What are the main findings?**

- TiO_2_ immobilization strategy influences the photocatalytic performance of BC–TiO_2_ nanocomposites.
- Vacuum filtration enables higher TiO_2_ loadings than agitation-assisted immobilization.
- Differences in photocatalytic performance occur without significant changes in TiO_2_ crystallinity or optical band gap.

**What are the implications of the main findings?**

- Immobilization strategy should be considered a design parameter for BC-supported photocatalysts.
- Photocatalytic performance depends on both catalyst loading and immobilization route.
- Simple aqueous immobilization methods enable tuning of BC–TiO_2_ photocatalytic performance.

**Abstract:**

Bacterial cellulose (BC) has been used as a renewable support for the development of photocatalytic nanocomposites due to its three-dimensional nanofibrillar network and large surface area. BC-based photocatalytic systems have been investigated for environmental applications, including the degradation of organic pollutants in wastewater. BC is commonly considered a passive scaffold. However, there is evidence suggesting that the strategy used to immobilize photocatalysts may influence the physicochemical characteristics of the resulting nanocomposites and their photocatalytic performance. This work investigates the effect of the TiO_2_ immobilization strategy on the photocatalytic performance of BC/TiO_2_ nanocomposites. Two simple immobilization approaches, namely agitation-assisted deposition and vacuum filtration-assisted deposition, were compared to evaluate how catalyst loading and immobilization routes influence the photocatalytic degradation of tartrazine as a model organic pollutant. The materials were characterized by FTIR, XRD, and UV–Vis, and their photocatalytic activity was evaluated through degradation kinetics. The results showed that photocatalytic performance is not only dependent on the amount of immobilized TiO_2_ but also on the immobilization strategy employed. FTIR tests revealed differences in the O-H stretching region, suggesting changes in the local chemical environment of cellulose surface hydroxyl groups, whereas XRD and UV–Vis analyses indicated that the crystalline structure and optical band gap of TiO_2_ remained essentially unchanged. These findings indicate that controlling the TiO_2_ immobilization strategy provides a simple strategy for tailoring the photocatalytic performance of BC-based nanocomposites.

## 1. Introduction

Semiconductor-based photocatalysis has emerged as a promising green technology for environmental remediation and sustainable energy production. Under light irradiation, semiconductor photocatalysts generate electron-hole pairs capable of promoting oxidation-reduction reactions responsible for pollutant degradation and hydrogen evolution. TiO_2_ is a commonly used photocatalyst because of its chemical stability, low cost, and high photocatalytic activity. For some applications, such as water treatment, the use of TiO_2_ is limited by the difficulty of recovering nanoparticles from treated water. Immobilization on solid supports has become an attractive strategy not only for improving catalyst recovery and reuse but also because the immobilization process itself may influence photocatalytic performance. Accordingly, this type of photocatalytic nanocomposite has received considerable attention [1,2,3].

Bacterial cellulose (BC) is a biopolymer synthesized by bacteria of the genus *Komagataeibacter*. It is formed by pure cellulose nanofibers forming a 3D network with high specific surface area, high mechanical strength, high stiffness, and excellent environmental compatibility [4,5]. These characteristics make BC an attractive support for photocatalysts. BC has been used as support for TiO_2_, ZnO, Ag_3_PO_4_, and g-C_3_N_4_ for applications including dye degradation, water purification, and photocatalytic hydrogen production [4,6].

A variety of strategies have been developed to immobilize photocatalysts on bacterial cellulose, including in situ synthesis, impregnation/adsorption, dip coating, vacuum-assisted filtration, and surface-functionalization-assisted immobilization approaches [7,8,9,10]. These methods have been explored to improve catalyst loading, nanoparticle dispersion, adhesion, stability, and photocatalytic efficiency, demonstrating that the immobilization procedure plays an important role in determining the final performance of BC-based photocatalytic systems. For example, Yang et al. [11] enhanced the immobilization of TiO_2_ nanoparticles on BC by introducing a polydopamine interlayer, resulting in improved nanoparticle anchoring, adsorption capacity, photocatalytic activity, and reusability. Recent reviews have highlighted that the choice of immobilization technique strongly influences catalyst distribution, coating morphology, adhesion, stability, and ultimately the operational performance of supported photocatalysts [12,13,14,15]. Nevertheless, most of these studies compare different material designs or introduce additional variables such as surface functionalization, binders, or in situ synthesis, making it difficult to isolate the specific influence of the immobilization strategy itself on the physicochemical characteristics and photocatalytic performance of BC-supported TiO_2_ nanocomposites.

Traditionally, BC has been considered a renewable support for catalyst immobilization [11]. Most studies have focused on improving photocatalytic performance by modifying the photocatalyst itself, for example, through compositional changes, doping, or heterojunction formation [16,17]. However, there is little information regarding how the immobilization strategy alone can influence the performance of BC-based photocatalytic nanocomposites. Different immobilization routes may produce nanocomposites with similar chemical composition but different spatial distribution of the photocatalyst and may generate different local chemical environments around cellulose surface hydroxyl groups. These differences may influence photocatalytic performance even when the intrinsic properties of the semiconductor remain unchanged.

The aim of this work was to investigate how the TiO_2_ immobilization strategy can be used as a design parameter to tailor the photocatalytic performance of BC–TiO_2_ nanocomposites. Agitation-assisted deposition and vacuum filtration-assisted deposition were compared using tartrazine degradation as a model reaction. FTIR, XRD, and UV–Vis spectroscopy were employed to evaluate the physicochemical characteristics of the resulting materials and to gain insight into the origin of the observed differences in photocatalytic performance.

## 2. Materials and Methods

### 2.1. Materials

Titanium dioxide (TiO_2_) nanoparticles (rutile phase, particle size <100 nm, ≥99.8% trace metals basis; Sigma-Aldrich, St. Louis, MO, USA) were used throughout the study. The same commercial TiO_2_ was used in all experiments to ensure that the immobilization strategy remained the only experimental variable. Tartrazine (Yellow No. 5) was supplied by Insuquímica S.A.C. (Lima, Peru). Purified bacterial cellulose membranes (BC-MD-0.2-S) were purchased from CelluloseLab (Fredericton, NB, Canada). Ultrapure water (18.2 MΩ·cm) was used to prepare all aqueous solutions. Unless otherwise stated, all chemicals were analytical grade and used without further purification.

### 2.2. Preparation of BC-TiO_2_ Nanocomposites

BC–TiO_2_ nanocomposites were prepared using two TiO_2_ immobilization routes: (i) agitation-assisted immobilization (AI) and (ii) vacuum filtration-assisted immobilization (VF). Prior to immobilization, TiO_2_ suspensions with different catalyst loadings (Table 1) were prepared in 50 mL of ultrapure water. The suspensions were sonicated for 30 min in an ultrasonic bath (Ultrasons-HD, 6 L, J.P. Selecta S.A., Barcelona, Spain) to ensure homogeneous nanoparticle dispersion [18,19]. BC membranes were cut into circular discs (70 mm diameter).

#### 2.2.1. Agitation-Assisted Immobilization (AI)

BC discs were immersed in the sonicated TiO_2_ suspension and maintained under magnetic stirring (140 rpm) for 60 min at room temperature to promote nanoparticle immobilization [20,21].

#### 2.2.2. Vacuum Filtration-Assisted Immobilization (VF)

BC discs were placed on a Büchner funnel using Whatman No. 1 filter paper (Cytiva, Marlborough, MA, USA) as support. The sonicated TiO_2_ suspension was then passed through the BC membrane under controlled vacuum until complete filtration was achieved [19,22].

#### 2.2.3. Post-Treatment

After immobilization, the nanocomposites were thoroughly washed with ultrapure water until no turbidity was observed in the washing solution, indicating the removal of loosely attached TiO_2_ nanoparticles [23,24]. The samples were subsequently stored in distilled water until characterization [21].

### 2.3. Characterization Tests

The morphology and elemental composition of the nanocomposites were analyzed by scanning electron microscopy (Quanta 650, FEI, Hillsboro, OR, USA) coupled with energy-dispersive X-ray spectroscopy (EDS) using an accelerating voltage of 15 kV and an acquisition time of 60 s. Thermogravimetric tests (30–800 °C) were performed using a TGA 4000 thermogravimetric analyzer (PerkinElmer, Waltham, MA, USA), under a N_2_ as inert atmosphere. Fourier transform infrared spectra were acquired in attenuated total reflectance mode (FTIR–ATR) using a PerkinElmer Spectrum 2 spectrometer (Waltham, MA, USA) over the range of 4000–450 cm^−1^ with a spectral resolution of 4 cm^−1^. X-ray diffraction (XRD) patterns were recorded using a Bruker D500 diffractometer (Bruker AXS GmbH, Karlsruhe, Germany) over a 2θ range of 20–80°. UV–Vis absorption spectra were recorded using a PerkinElmer Lambda 850 spectrophotometer (PerkinElmer, Waltham, MA, USA). The optical band-gap energy of the nanocomposites was estimated using the Tauc method.

### 2.4. Photocatalytic Activity

Photocatalytic activity was evaluated using tartrazine as a model organic pollutant. After immobilization, the BC–TiO_2_ membranes (70 mm diameter) were cut into circular discs (10 mm diameter) to fit the sample vials of the photoreactor. The discs were individually immersed in 7.5 mL of tartrazine solution (50 mg L^−1^, pH 5.5). The experiments were performed in an EvoluChem™ Photoredox device equipped with a 365 nm UV light source (HepatoChem, Inc., Beverly, MA, USA). Prior to irradiation, the samples were kept in the dark for 20 min to establish adsorption–desorption equilibrium. Subsequently, the samples were irradiated for 120 min under continuous stirring. Aliquots were collected every 30 min, and the residual tartrazine concentration was determined by measuring the absorbance at 426 nm using UV–Vis spectroscopy. For comparison, photocatalytic experiments were also carried out using pristine TiO_2_ suspensions containing catalyst loadings equivalent to those immobilized in each BC–TiO_2_ nanocomposite.

### 2.5. Photocatalytic Kinetic Analysis

The degradation kinetics of tartrazine were analyzed using the Langmuir–Hinshelwood pseudo-first-order kinetic model. The normalized concentration (C/C_0_) was plotted as a function of irradiation time, and the apparent reaction rate constant (*k_app_*) was determined from the linearized kinetic equation.

Use of Generative Artificial Intelligence. During the preparation of this manuscript, ChatGPT (OpenAI, GPT-5.5) was used to assist with drafting and refining scientific text in English and improving manuscript organization.

## 3. Results and Discussion

**Figure 1:**
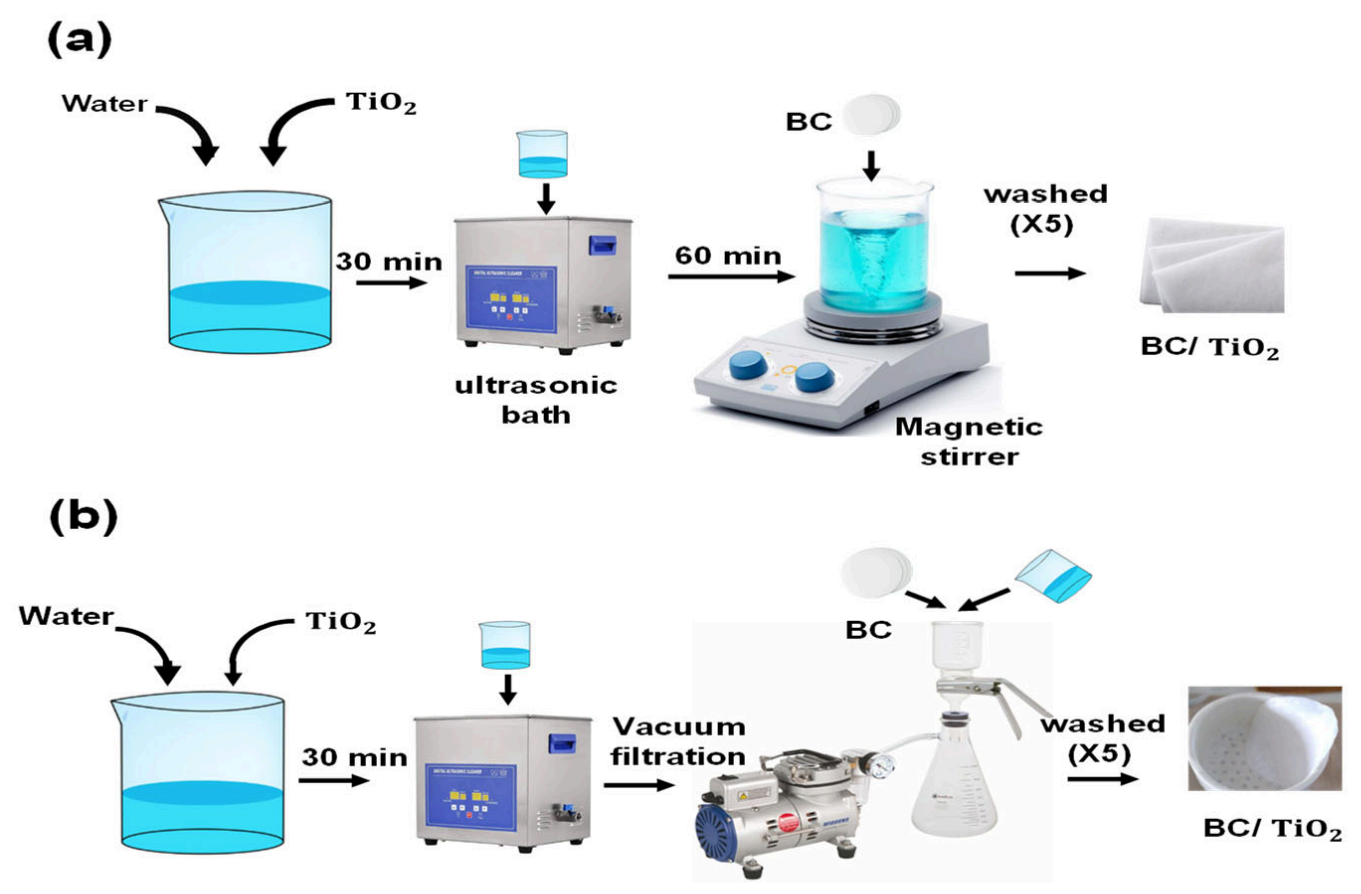
Schematic illustration of the two TiO_2_ immobilization routes used to prepare BC–TiO_2_ nanocomposites. (**a**) In the agitation-assisted immobilization (AI) route, BC membranes were immersed in a sonicated TiO_2_ suspension under magnetic stirring. (**b**) In the vacuum filtration-assisted immobilization (VF) route, the same TiO_2_ suspension was passed through the BC membrane under reduced pressure. Both approaches employed identical precursor suspensions, differing only in the nanoparticle immobilization route.

To investigate the influence of the immobilization strategy, two BC–TiO_2_ nanocomposites were prepared using different TiO_2_ deposition routes while keeping all other processing variables constant (Figure 1). In the first route, TiO_2_ nanoparticles were immobilized by immersing the BC membrane in the sonicated suspension under magnetic stirring (agitation-assisted immobilization, AI), whereas in the second route the suspension was passed through the BC membrane by vacuum filtration (vacuum filtration-assisted immobilization, VF). These two strategies impose different routes for incorporating TiO_2_ into the BC network and were selected to evaluate whether the immobilization strategy alone influences the physicochemical characteristics and photocatalytic performance of BC-supported TiO_2_ nanocomposites.

Thermogravimetric analysis (TGA) was used to estimate the amount of immobilized TiO_2_ from the residual mass remaining after complete thermal decomposition of BC, following methodologies commonly adopted for cellulose-supported TiO_2_ nanocomposites [25]. The presence of Ti on the BC surface was confirmed by SEM–EDX analysis (Figure S1), supporting the assumption that the residual mass corresponds predominantly to retained TiO_2_. As summarized in Table 2, the agitation-assisted immobilization route produced TiO_2_ loadings ranging from 7.575 to 23.277 wt%, whereas the vacuum filtration route yielded significantly higher values ranging from 16.930 to 36.002 wt%. These results demonstrate that vacuum filtration was more effective for immobilizing TiO_2_ onto the bacterial cellulose substrate under the investigated conditions. The higher TiO_2_ loading achieved by vacuum filtration is consistent with previous reports describing bacterial cellulose as a three-dimensional hydroxyl-rich scaffold that promotes TiO_2_ nucleation and immobilization while minimizing aggregation [26].

Tartrazine degradation under UV irradiation was used as a model reaction to investigate how different TiO_2_ immobilization strategies affect the photocatalytic performance of BC-based nanocomposites. For comparison, tartrazine degradation was also evaluated using pristine TiO_2_ suspensions containing catalyst loadings equivalent to those immobilized in each BC-based nanocomposite. Although suspended and immobilized photocatalytic systems are intrinsically different, pristine TiO_2_ suspensions were used as a reference to assess the relative preservation of photocatalytic performance after immobilization.

**Figure 2:**
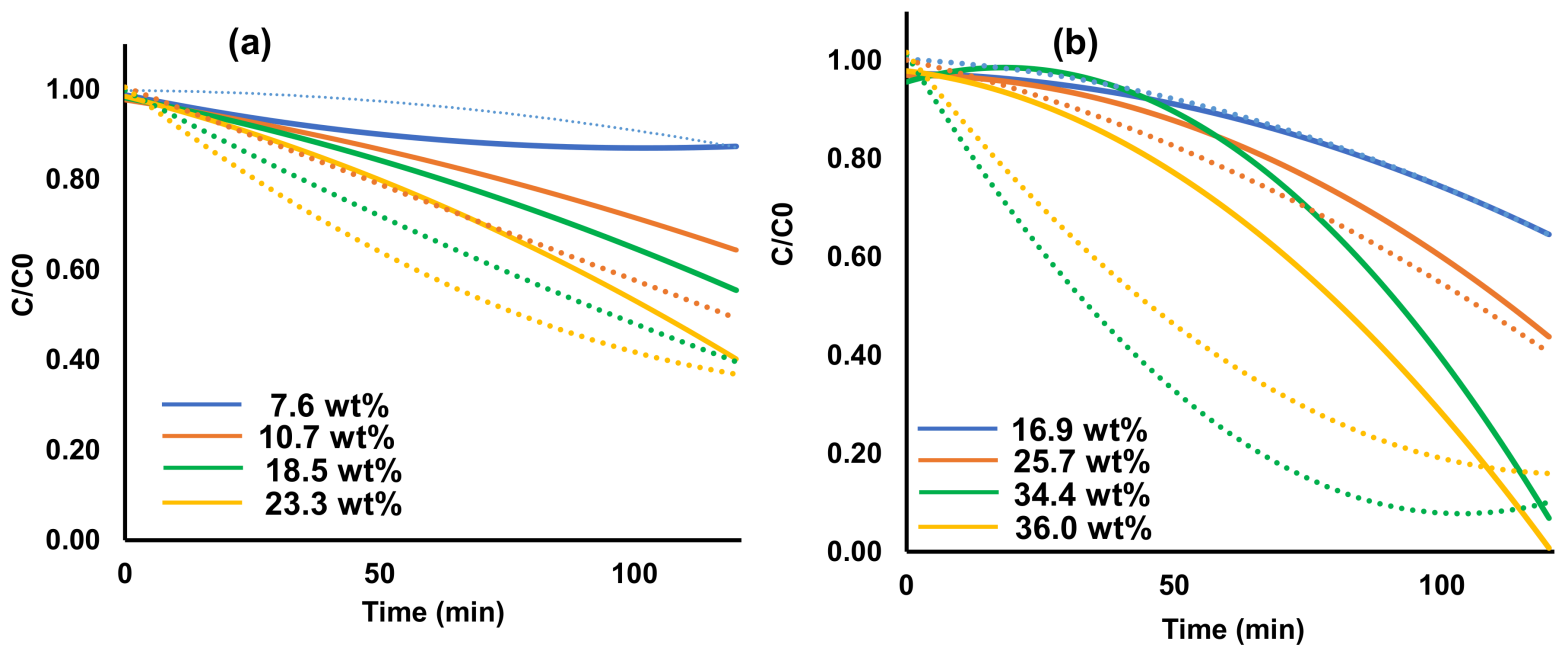
Photocatalytic degradation kinetics of tartrazine under UV irradiation obtained with BC–TiO_2_ nanocomposites prepared by different immobilization routes. (**a**) Agitation-assisted immobilization. (**b**) Vacuum filtration-assisted immobilization. Solid lines represent BC-supported TiO_2_ nanocomposites, whereas dotted lines correspond to pristine TiO_2_ suspensions containing equivalent TiO_2_ loadings. The concentration of tartrazine is expressed as the normalized concentration (C/C_0_).

Figure 2 shows that the immobilization strategy had an influence on the photocatalytic degradation kinetics of tartrazine. For agitation-assisted immobilization (Figure 2a), all BC-supported TiO_2_ nanocomposites exhibited slower degradation kinetics than pristine TiO_2_ suspensions containing equivalent catalyst loadings. The immobilization assisted by agitation reduced the intrinsic photocatalytic activity of the semiconductor. A different behavior was observed for vacuum filtration-assisted immobilization (Figure 2b). At the two lowest TiO_2_ loadings (16.9 and 25.7 wt%), the BC-supported nanocomposites also exhibited slightly lower photocatalytic activity than the corresponding pristine TiO_2_ suspensions. However, at higher TiO_2_ loadings (34.4 and 36.0 wt%), the VF nanocomposites matched or exceeded the performance of pristine TiO_2_, indicating that the vacuum filtration strategy was able to preserve, and even enhance, the overall photocatalytic performance at high catalyst loadings. It is worth noting that, at comparable TiO_2_ contents, the AI nanocomposites tended to exhibit slightly higher photocatalytic activity than the corresponding VF nanocomposites. Nevertheless, the maximum TiO_2_ loading achieved by agitation-assisted immobilization (23.3 wt%) was substantially lower than that obtained by vacuum filtration (36.0 wt%). Consequently, only the VF strategy enabled the preparation of high-loading nanocomposites capable of outperforming pristine TiO_2_ suspensions. Finally, the pristine TiO_2_ suspension containing 36.0 wt% catalyst exhibited a slightly lower final degradation than that containing 34.4 wt%, suggesting that an optimum catalyst loading may have been exceeded. Such behavior has been widely reported in slurry photocatalysis and is commonly attributed to increased light scattering (optical screening) and particle agglomeration at high catalyst concentrations, which reduce the effective utilization of incident radiation and the available active surface area [27,28].

**Figure 3:**
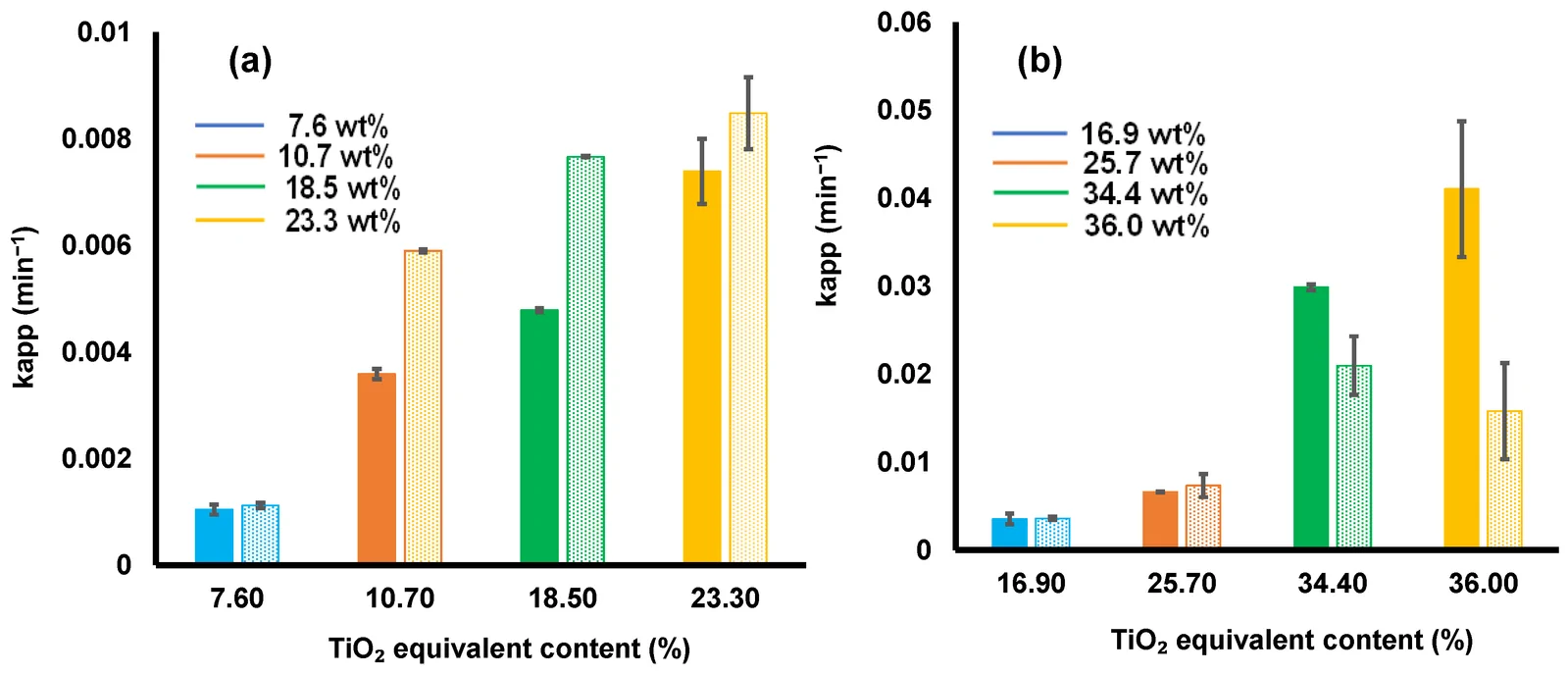
Apparent pseudo-first-order rate constants (k_app_) obtained from Langmuir–Hinshelwood kinetic analysis of tartrazine photodegradation using BC–TiO_2_ nanocomposites prepared by (**a**) agitation-assisted immobilization and (**b**) vacuum filtration-assisted immobilization. Solid bars represent BC-supported TiO_2_ nanocomposites, whereas patterned bars correspond to pristine TiO_2_ suspensions containing equivalent TiO_2_ loadings included for comparison.

To quantify the differences observed in the degradation profiles, the apparent kinetic constants (*k*_app_) were calculated. *K*_app_ was obtained by fitting the degradation data to a pseudo-first-order kinetic model according to the Langmuir–Hinshelwood approximation (Figure S2). This model is widely used to describe heterogeneous photocatalytic degradation at low pollutant concentrations and provides a consistent basis for comparing the apparent degradation rates of different photocatalytic systems [29]. For agitation-assisted immobilization, all BC-supported nanocomposites exhibited lower *k*_app_ values than pristine TiO_2_ at equivalent catalyst loadings (Figure 3a), indicating that immobilization under these conditions reduced the overall photocatalytic reaction rate. In contrast, nanocomposites prepared by vacuum filtration showed *k*_app_ values comparable to, or higher than, those obtained for pristine TiO_2_, particularly at higher catalyst loadings (Figure 3b).

To quantify the effect of the immobilization strategy on photocatalytic performance, a Relative Photocatalytic Activity Factor (RPAF) was defined as the ratio between the apparent kinetic constant of the BC-supported TiO_2_ nanocomposite and that of the pristine TiO_2_ suspension containing an equivalent catalyst loading:

(1) $$RPAF=\frac{k_{app,BC-{TiO}_{2}}}{k_{app,pristine{TiO}_{2}\;}}$$

An RPAF value equal to unity indicates that immobilization preserves the intrinsic photocatalytic activity of TiO_2_, whereas values below and above unity indicate activity losses and activity enhancement after immobilization, respectively.

**Figure 4:**
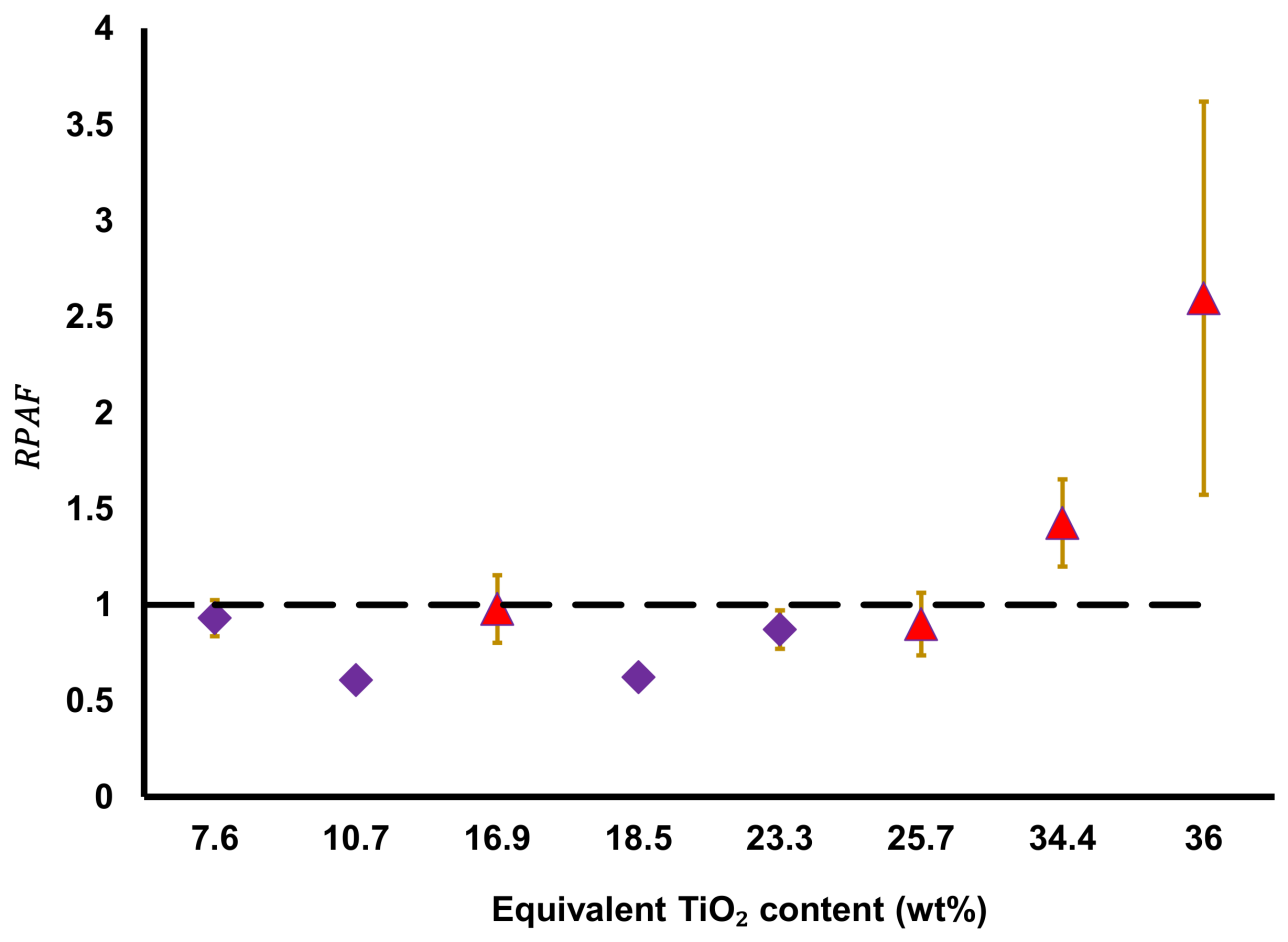
Relative Photocatalytic Activity Factor (RPAF) of BC-supported TiO_2_ nanocomposites prepared by agitation-assisted immobilization (◆) and vacuum-assisted filtration (▲) as a function of the equivalent TiO_2_ loading. The dashed horizontal line (RPAF = 1) indicates identical photocatalytic activity to pristine TiO_2_.

The calculated RPAF values (Figure 4) show different behaviors for the two immobilization routes. Samples prepared by agitation-assisted immobilization exhibited RPAF values below unity, indicating that this immobilization strategy reduced the effective photocatalytic activity of TiO_2_ over the entire range of catalyst loadings. The immobilized photocatalysts never reached the performance of the corresponding pristine TiO_2_ suspensions.

In contrast, vacuum filtration immobilization produced a progressive increase in RPAF with increasing TiO_2_ loading. At low catalyst loadings, the photocatalytic activity was comparable to that of pristine TiO_2_ (RPAF ≈ 1), whereas at higher loadings the RPAF exceeded unity, demonstrating that the BC-supported photocatalyst outperformed the corresponding pristine TiO_2_ suspension. The operational stability of the BC–TiO_2_ nanocomposites was further assessed through four consecutive photocatalytic cycles (Figure S3). Both immobilization strategies retained photocatalytic activity throughout the reuse tests under continuous irradiation and stirring, with the vacuum filtration nanocomposites maintaining higher degradation efficiencies during all cycles. These results indicate that the photocatalytic behavior observed in the single-cycle experiments was preserved during repeated operation.

**Figure 5:**
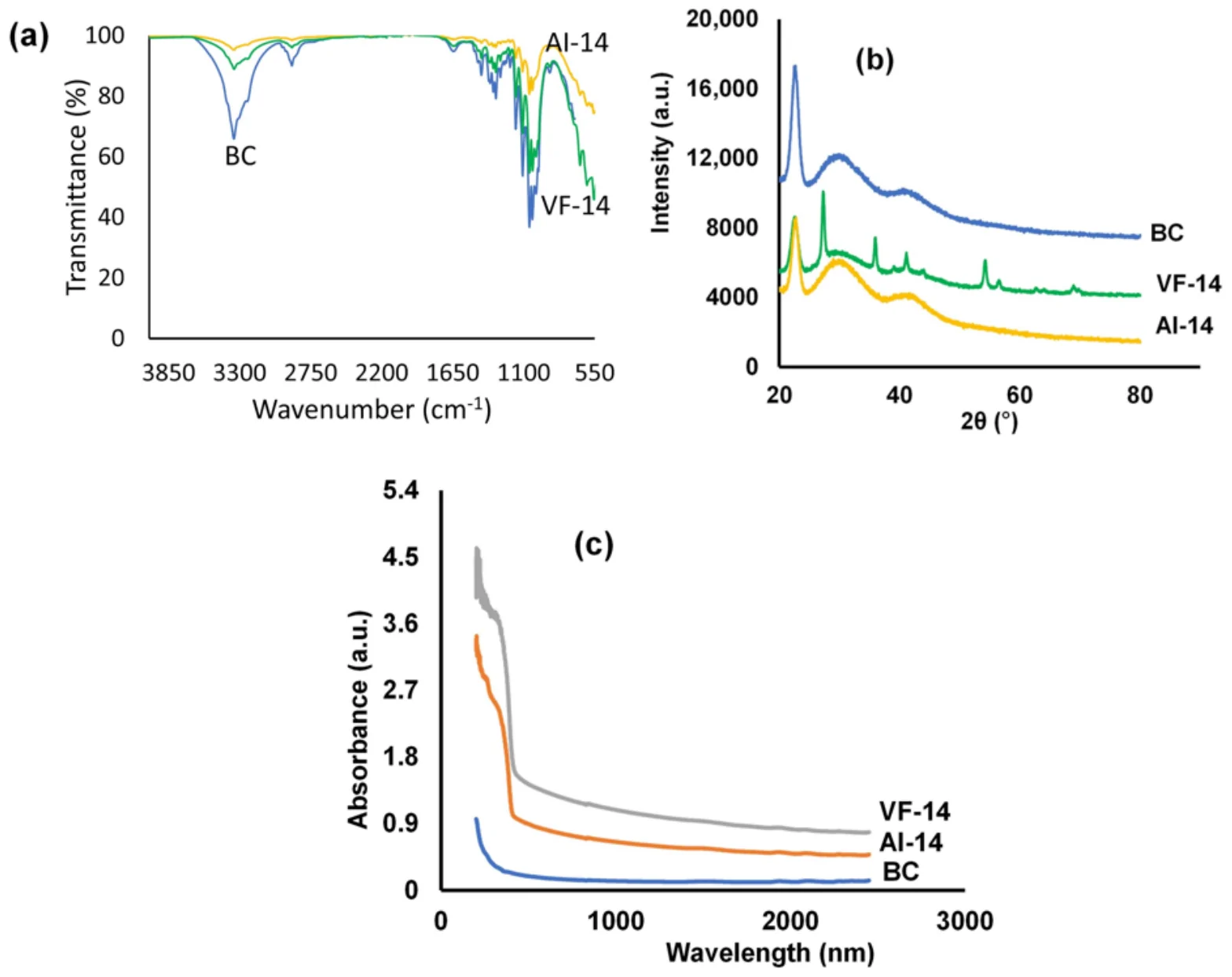
Representative (**a**) FTIR spectra, (**b**) XRD patterns, and (**c**) UV–Vis absorption spectra of pristine bacterial cellulose (BC) and BC-supported TiO_2_ nanocomposites prepared by agitation-assisted immobilization (AI-14) and vacuum-assisted filtration (VF-14).

The distinct kinetic behavior, together with the RPAF values, demonstrates that the TiO_2_ immobilization strategy determines not only the amount of photocatalyst incorporated into the BC network but also the resulting photocatalytic performance. These observations suggest that the immobilization strategy modifies the physicochemical characteristics of the BC–TiO_2_ nanocomposites that ultimately influence their photocatalytic behavior. To identify these characteristics, the materials were subsequently analyzed by FTIR, XRD, and UV–Vis spectroscopy. Figure 5 shows representative FTIR, XRD, and UV–Vis spectra of pristine BC and BC–TiO_2_ nanocomposites prepared by agitation-assisted (AI) and vacuum filtration-assisted (VF) immobilization. The FTIR spectra of pristine BC display the characteristic cellulose Iβ absorption bands, including the broad O–H stretching band (~3340 cm^−1^), C–H stretching (~2900 cm^−1^), and the C–O–C and C–O vibrations in the fingerprint region [30,31]. Pristine TiO_2_ exhibits the characteristic Ti–O/Ti–O–Ti vibrations below 800 cm^−1^ (Figure S4). Both BC–TiO_2_ nanocomposites retain the characteristic absorption bands of BC while exhibiting the characteristic Ti–O/Ti–O–Ti vibrations below 800 cm^−1^ (Figure S4), confirming successful incorporation of TiO_2_. In addition, slight broadening and a small shift in the O–H stretching band toward lower wavenumbers, together with a shoulder near 1050 cm^−1^, were observed after TiO_2_ immobilization [22,32]. XRD patterns revealed the characteristic diffraction peaks of cellulose Iβ (2θ ≈ 22.5°, 14.5° and 16.6°) and rutile TiO_2_ (2θ ≈ 27.4°, 36.1°, 39.2°, 41.2°, 54.3° and 56.6°) in both nanocomposites, indicating that TiO_2_ was successfully incorporated without altering the crystalline structure of BC [30,31]. Finally, UV–Vis spectra showed negligible absorption for pristine BC, whereas both AI and VF nanocomposites exhibited strong absorption in the UV region (λ < 400 nm), characteristic of the O^2−^ → Ti^4+^ charge-transfer transitions of TiO_2_ [21].

The results presented in Figure 5 provide insight into the physicochemical characteristics of the BC–TiO_2_ interface generated by the two immobilization strategies. The broadening and slight shift in the O–H stretching band, together with the shoulder around 1050 cm^−1^, have been previously associated with changes in hydrogen-bonding environments and interfacial interactions in cellulose/TiO_2_ composites [22,32]. Similar spectral changes have been reported in BC–TiO_2_ and cellulose–TiO_2_ hybrid materials prepared by different immobilization approaches [21]. Although FTIR does not provide direct evidence of the nature of the interfacial interactions, the observed spectral differences indicate that the immobilization strategy modifies the local chemical environment of cellulose surface hydroxyl groups. Such differences may be associated with the distinct photocatalytic performances observed for the two immobilization strategies.

**Figure 6:**
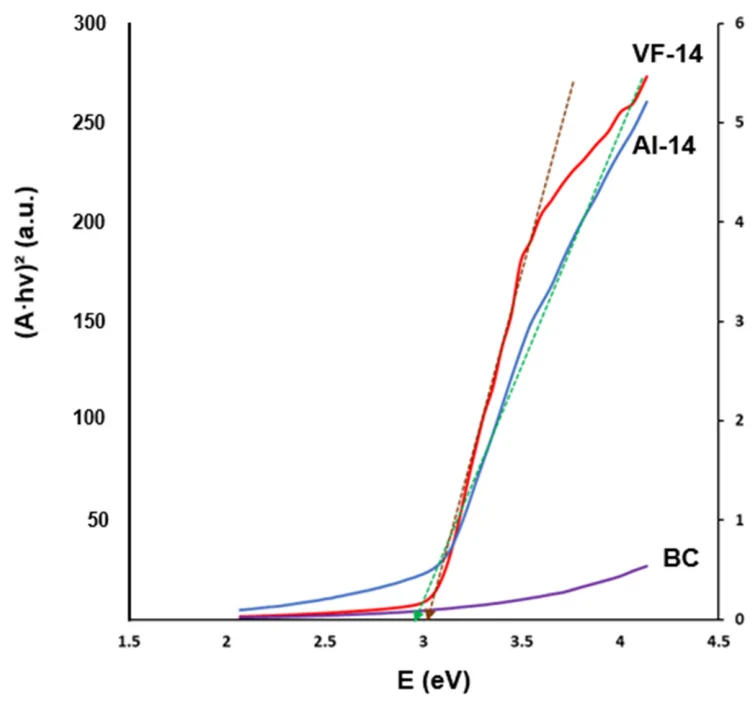
Tauc plots obtained from the UV–Vis absorption spectra of pristine bacterial cellulose (BC) and BC–TiO_2_ nanocomposites prepared by agitation-assisted immobilization (AI) and vacuum filtration-assisted immobilization (VF). The optical band-gap energy (Eg) was estimated by extrapolating the linear region of the plots using the indirect transition model (n = ½) for rutile TiO_2_. The dotted lines represent the linear extrapolation of the selected regions used to estimate Eg from their intercept with the energy axis.

The optical properties of the BC–TiO_2_ nanocomposites were further evaluated using Tauc plots derived from the UV–Vis absorption spectra (Figure 6). The Tauc method is widely employed to estimate the optical band-gap energy (Eg) of semiconductors by extrapolating the linear region of the relationship between the absorption coefficient and photon energy. Since rutile TiO_2_ is an indirect semiconductor, the band-gap energy was determined using the indirect transition model (n = ½) [33]. The estimated Eg values ranged from 3.00 to 3.15 eV for the AI nanocomposites and from 2.95 to 3.05 eV for the VF nanocomposites. Given the inherent limitations of the Tauc method for accurately resolving small band-gap differences in polycrystalline semiconductors [34], the variations in the Eg values calculated in this study should not be considered significant. These results indicate that the immobilization process did not substantially modify the optical properties of TiO_2_. Therefore, the different photocatalytic performances observed for the AI and VF nanocomposites cannot be attributed primarily to changes in their optical absorption properties.

These results indicate that the two immobilization strategies produce BC–TiO_2_ nanocomposites with similar crystalline structure and optical properties but different local chemical environments at the cellulose surface. These observations suggest that the photocatalytic performance is influenced by physicochemical differences introduced during the immobilization process rather than by significant modifications of the intrinsic properties of TiO_2_.

## 4. Conclusions

Although both immobilization methods employed the same photocatalyst under identical preparation conditions, agitation-assisted immobilization and vacuum filtration produced nanocomposites with different TiO_2_ loadings and distinct photocatalytic behaviors. Vacuum filtration enabled higher TiO_2_ incorporation into the BC network and produced the highest photocatalytic performance, whereas agitation-assisted immobilization exhibited slightly higher photocatalytic activity at comparable TiO_2_ loadings but was limited in the maximum amount of TiO_2_ that could be immobilized. Both the catalyst loading and the immobilization strategy contribute to the final photocatalytic performance of BC-supported TiO_2_ nanocomposites.

FTIR analysis revealed differences in the chemical environment of cellulose surface hydroxyl groups between the two immobilization routes, while XRD and UV–Vis analyses indicated that the crystalline structure and optical band gap of TiO_2_ remained essentially unchanged after immobilization. These findings suggest that the differences in photocatalytic performance are associated with changes introduced during the immobilization process rather than with modifications of the intrinsic structural or optical properties of TiO_2_. Future studies should investigate the reactive oxygen species involved in the degradation process and evaluate the influence of the immobilization strategy under visible-light irradiation.

This work demonstrates that the TiO_2_ immobilization strategy can be used as an effective design parameter for tailoring the performance of BC-supported photocatalysts using simple aqueous processing methods. The results provide useful guidelines for the design of renewable cellulose-based photocatalytic materials for environmental remediation.

## Figures and Tables

**Table 1:** BC-TiO_2_ samples prepared using two different immobilization routes.

Immobilization Route	TiO_2_ Suspension (g/50 mL)	Sample Code
Agitation-assisted immobilization (AI)	0.010305	AI-01
	0.03435	AI-03
	0.0687	AI-07
	0.1374	AI-14
Vacuum filtration-assisted immobilization (VF)	0.010305	VF-01
	0.03435	VF-03
	0.0687	VF-07
	0.1374	VF--14

**Table 2:** TiO_2_ amount (wt%) immobilized on BC, according to TGA measurements.

Immobilization Route	TiO_2_ wt%	Sample Code
Agitation-assisted immobilization (AI)	7.575	AI-01
	10.682	AI-03
	18.535	AI-07
	23.277	AI-14
Vacuum filtration-assisted immobilization (VF)	16.930	VF-01
	25.744	VF-03
	34.390	VF-07
	36.002	VF--14

## Data Availability

The original contributions presented in this study are included in the article/Supplementary Material. Further inquiries can be directed to the corresponding author.

## Supplementary Materials

The following supporting information can be downloaded at https://www.mdpi.com/article/10.3390/ma19173575/s1.
