# Supplementary material for: Influence of TiO2 Immobilization Strategy on BC–TiO2 Nanocomposite Photocatalytic Performance
Source: Materials (Basel). 2026 Aug 23;19(17):3575. doi: 10.3390/ma19173575 (PMC13566674; doi:10.3390/ma19173575)
Supplement: Supplementary file 1 [file materials-19-03575-s001.zip › materials-4477584-supplementary.pdf]

Supplementary Material

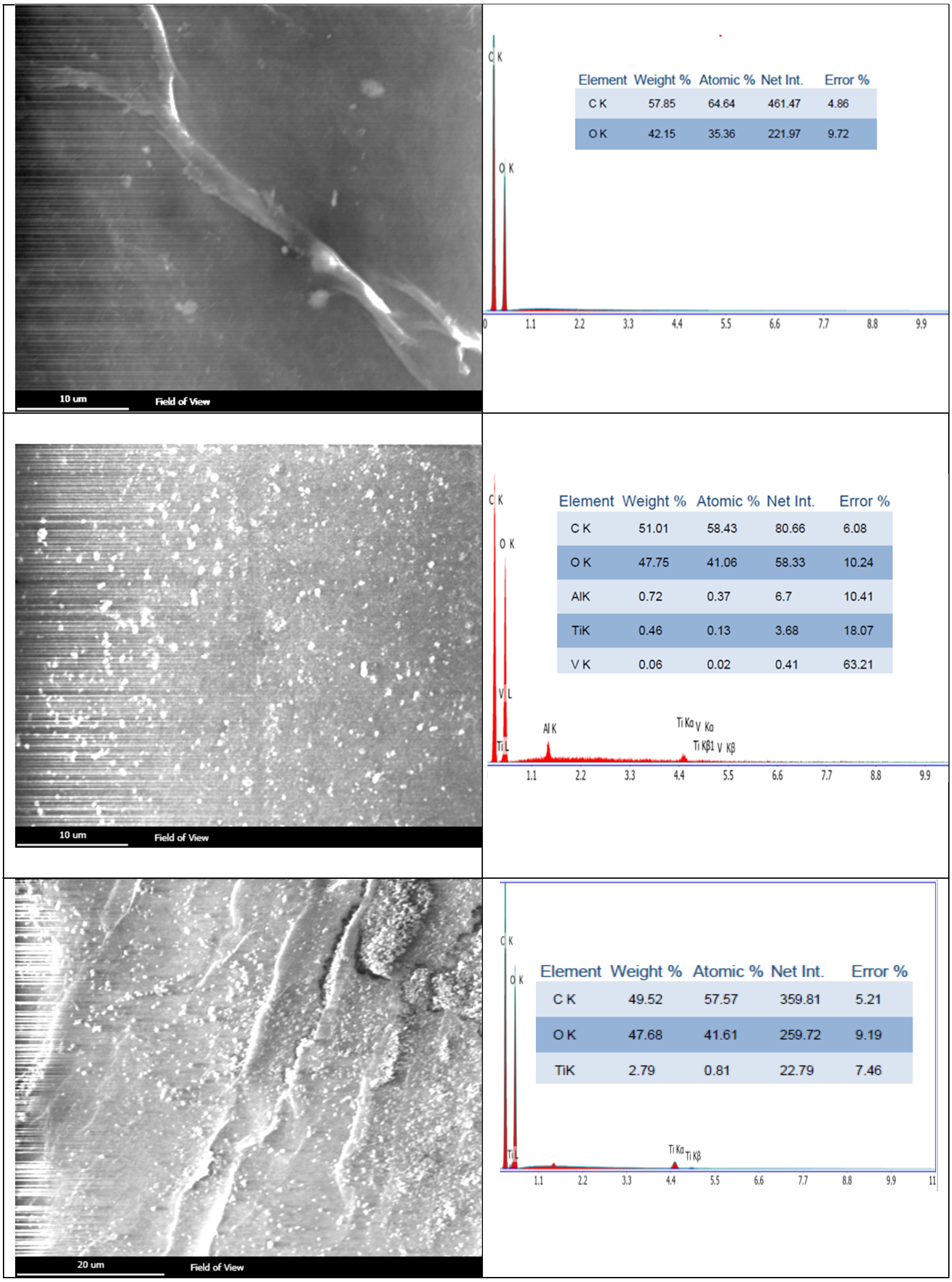

Figure S1. SEM micrographs and EDX spectra of the bacterial cellulose (BC) membranes before and after TiO_2 immobilization. (a) SEM image of pristine BC. (b) EDX spectrum of pristine BC. (c) SEM image of the BC–TiO_2 nanocomposite prepared by agitation-assisted immobilization (AI). (d) EDX spectrum of the BC–TiO_2 (AI) nanocomposite. (e) SEM image of the BC–TiO_2 nanocomposite prepared by vacuum filtration-assisted immobilization (VF). (f) EDX spectrum of the BC–TiO_2 (VF) nanocomposite.

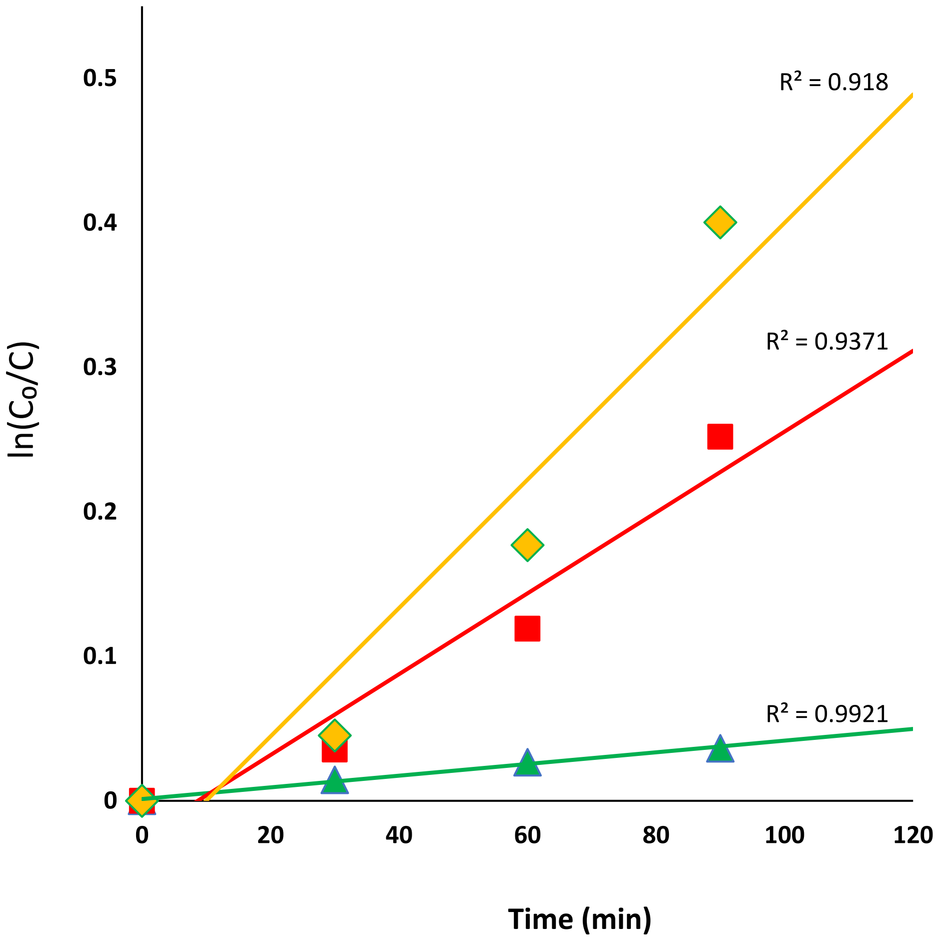

Figure S2. Pseudo-first-order kinetic fitting ($\ln(C_0/C)$ versus time) for Al-03 (Δ), Al-07 (◊), and Al-14 (□) samples.

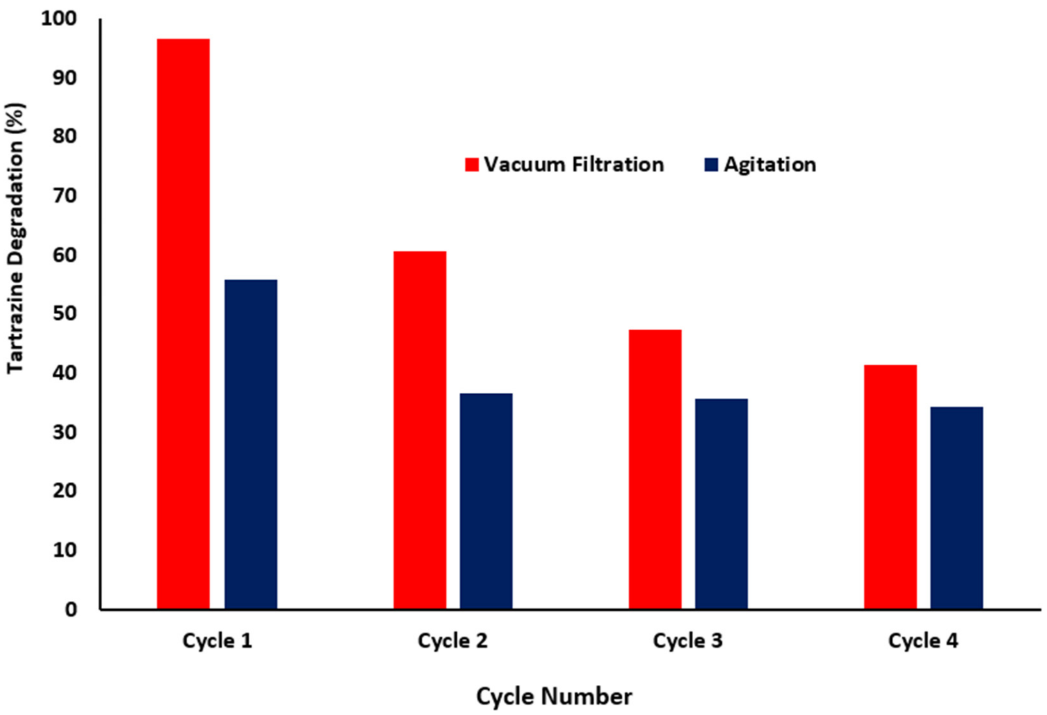

Figure S3. Operational stability of BC–TiO_2 nanocomposites prepared by agitation-assisted immobilization (AI) and vacuum filtration-assisted immobilization (VF) during four consecutive photocatalytic degradation cycles of tartrazine under UV irradiation (365 nm). After each 2 h photocatalytic cycle, the nanocomposites were recovered, rinsed thoroughly with deionized water to remove residual dye and reaction products, and reused under identical experimental conditions. The degradation efficiency after each cycle was used to evaluate the operational stability and reusability of the immobilized photocatalysts.

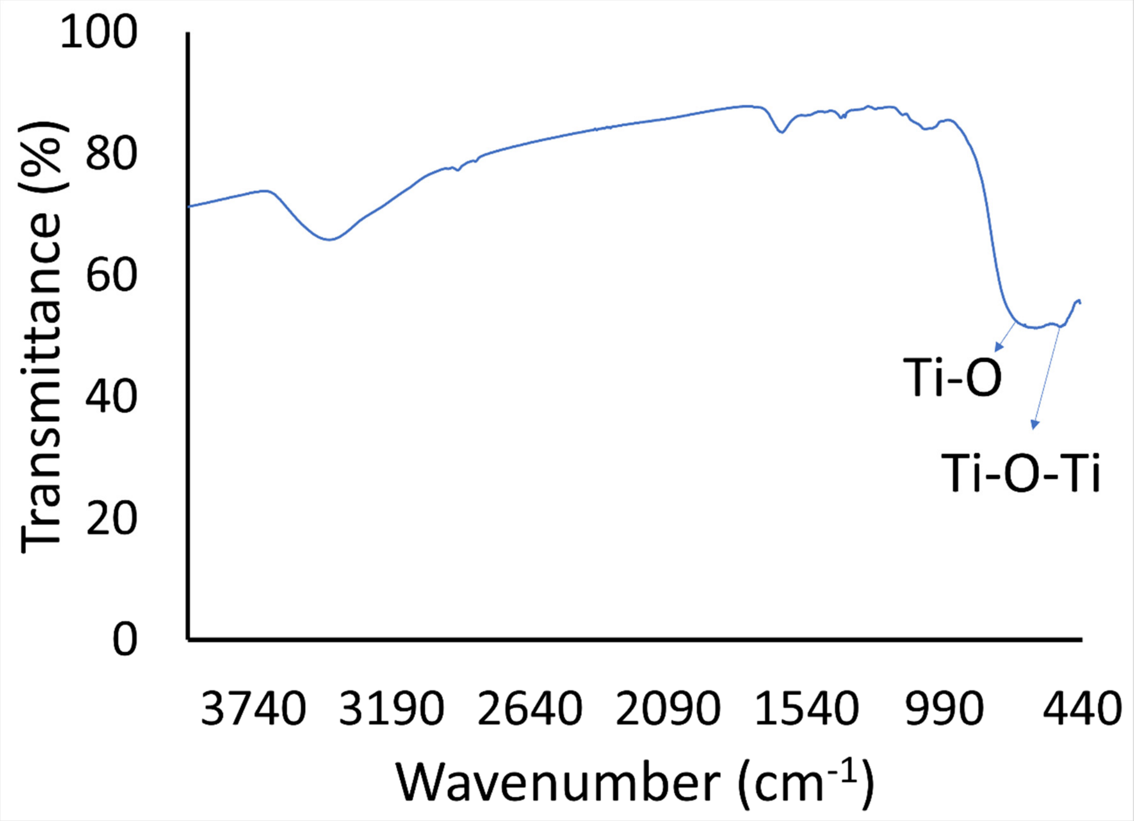

Figure S4. Representative FTIR spectrum of a pristine TiO_2 sample.
